# Supplementary material for: Exploring the phenotypic consequences of tissue specific gene expression variation inferred from GWAS summary statistics
Source: Nat Commun. 2018 May 8;9:1825. doi: 10.1038/s41467-018-03621-1 (PMC5940825; doi:10.1038/s41467-018-03621-1)
Supplement: Supplementary file 1 — Supplementary Information [file 41467_2018_3621_MOESM1_ESM.pdf]

Supplementary Notes

Supplementary Note 1: S-PrediXcan vs. PrediXcan in simulated data

We measure performance as the ability of S-PrediXcan to infer accurate PrediXcan results. Differences arise mostly because of LD differences between reference and study sets. We use genotype data from different populations from 1000G to assess the robustness to large differences between reference and study sets.

First we simulated normally distributed phenotype (under the null hypothesis of no genetic effect). We use prediction models trained on Depression Genes and Network's (DGN) Whole Blood data set [1,2] downloaded from PredictDB (http://predictdb.org). For genotypes we used three ancestral subsets of the 1000 Genomes project: Africans (n=661), East Asians (n=504), and Europeans (n=503). Each set was taken in turn as reference and study set yielding a total of 9 combinations as shown in Fig. 2a. For each population combination, we computed PrediXcan association results for the simulated phenotype and compared them with results generated using S-PrediXcan in a scatter plot. In this manner we assess the effect of ancestral differences between study and reference sets.

As expected, when the study and reference sets are the same, the concordance between PrediXcan and S-PrediXcan is almost 100%, whereas for sets of different ancestral origin the R^2 drops a few percentage points, with the biggest loss (down to 85%) when the study set is African and the reference set is Asian. This confirms that our formula works as expected and that the approach is robust to substantial differences between study and reference sets.

Supplementary Note 2: S-PrediXcan vs. PrediXcan in real data (cellular phenotype)

Next we tested with an actual cellular phenotype - intrinsic growth. This phenotype was computed based on multiple growth assays for over 500 cell lines from the 1000 Genomes project [3]. We used a subset of values for European (EUR), African (AFR), and Asian (EAS) individuals.

We compared Z-scores for intrinsic growth generated by PrediXcan and S-PrediXcan for different combinations of reference and study sets, using whole blood prediction models trained in the DGN cohort. The results are shown in Fig. 2b. As with our simulation study, the S-PrediXcan results closely match the PrediXcan results. Again, the best concordance occurs when reference and study sets share similar continental ancestry while differences in population slightly reduce concordance. Compared to the plots for the simulated phenotypes, the diagonal concordance is slightly lower than 1. This is due to the fact that more individuals were

included in the reference set than in the study set, thus the study and reference sets were not identical for S-PrediXcan.

Supplementary Note 3: S-PrediXcan vs. PrediXcan in disease phenotypes from WTCCC

We show the comparison of PrediXcan and summary-PrediXcan results for two diseases: Bipolar Disorder (BD) and Type 1 Diabetes (T1D) from the WTCCC in Fig. 2c. Concordance between PrediXcan and Summary-PrediXcan is over 99% for both diseases (BD $R^2 = 0.996$ and T1D $R^2 = 0.995$). The very small discrepancies are explained by differences in allele frequencies and LD between the reference set (1000 Genomes) and the study set (WTCCC).

It is worth noting that the PrediXcan results for diseases were obtained using logistic regression whereas Summary-PrediXcan formula is based on linear regression. As observed before [4], when the number of cases and controls are relatively well balanced (roughly, at least 25% of a cohort are cases or controls), linear regression approximation yields very similar results to logistic regression. This high concordance also shows that the approximation of dropping the factor $\sqrt{\frac{1-R_l^2}{1-R_g^2}}$ does not significantly affect the results.

Supplementary Note 4: PrediXcan Rationale

There is a large amount of evidence indicating that a substantial portion of genetic effect on phenotype is mediated via alteration of gene expression levels. Studies of enrichment of expression quantitative trait loci (eQTLs) among trait-associated variants [5–7] show the importance of this relationship.

Given the success of GWAS approaches, large-scale GWAS/GWAMA efforts with ever increasing sample sizes are underway, which will be able to detect variants of smaller effects sizes. QTL studies, on the other hand, are currently limited to smaller sample sizes.

To take advantage of GWAS and QTL study data, PrediXcan was designed to test the mediating molecular trait's effect on a phenotype [8]. Its purpose is to identify trait-associated genes using genetically predicted molecular traits such as gene expression. PrediXcan uses independent QTL studies to train prediction models of the molecular trait. Using these models, PrediXcan imputes gene expression levels in the GWAS study cohort, and then correlates the association of the predicted gene expression levels to the phenotype of interest.

This means that PrediXcan has the following features [8]:

- Reduced testing burden. By performing gene-level tests, the computational burden is significantly reduced when compared to single variant tests. $20,000$ gene-level tests are need at most, whereas $10,000,000$ test may be needed in a single-variant analysis.
- Direction of effect. PrediXcan provides the direction of effect of the association, so that potential targets for either down-regulation or up-regulation can be identified.
- Reduced reverse causality problems. Drug treatment or disease status may affect a molecular trait without modifying germline genomic variation. Germline is not affected by disease and prediction models are built in independent cohorts such that when there is a causal relationship the direction goes mostly from the predicted gene expression to the disease.
- Does not claim causality. PrediXcan may yield significant association in cases where the causal variant for the gene expression and the causal variant for the phenotype are different but are in LD. This can be mitigated using colocalization measures to filter out these LD-contaminated associations. Also, PrediXcan cannot distinguish between causal relationship and pleiotropy. For example, if the same causal SNP affects the expression level of two genes, where one is causal and the other one is not, PrediXcan will not be able to distinguish between them.
- Detection of aggregates of small effects. In cases when multiple causal variants affect gene expression levels, it may happen that the individual SNP association does not reach genome-wide significance but the combined multi-SNP effect does clear the threshold because of the combination of reduced multiple testing burden and increased effect size of the multi-SNP combination.

In summary, one of the main benefits of PrediXcan (and S-PrediXcan) over GWAS is that it returns genes rather than SNPs since much more is known about the function of genes. Another important benefit is the increased power because of the reduced multiple testing burden and the potentially larger effect sizes of the imputed genes.

Supplementary Note 5: Author Contribution

A.N.B. Contributed to S-PrediXcan software development. Executed S-PredixCan runs on the GWAS traits. Developed framework for comparing PrediXcan and S-PrediXcan in simulated, cellular and WTCCC phenotypes.

Ran COLOC and SMR. Developed gene2pheno.org database and web dashboard. Contributed to the main text, supplement, figures and analyses.

S.P.D. Performed the GTEx model training. Ran the GERA GWAS. Contributed to the main text.

J.M.T. Contributed to the main text.

J.Z. contributed to the figures and predictdb.org resource.

E.S.T. contributed to S-PrediXcan software.

H.E.W. contributed to the main text and analysis.

K.P.S. ran PrediXcan on WTCCC data and contributed to the analysis.

R.B. contributed to the main text and figures.

T.G. Ran imputation of GERA genotypes.

T.E. contributed to the analysis.

D.N. contributed to the main text and analysis.

N.J.C. contributed to the analysis.

H.K.I. conceived the method, supervised the project, performed analysis, contributed to the main text, supplement, and figures.

The GTEx consortium authors contributed in the collection, gathering and processing of GTEx study data used for training transcriptome prediction models and running COLOC and SMR.

Supplementary Figures

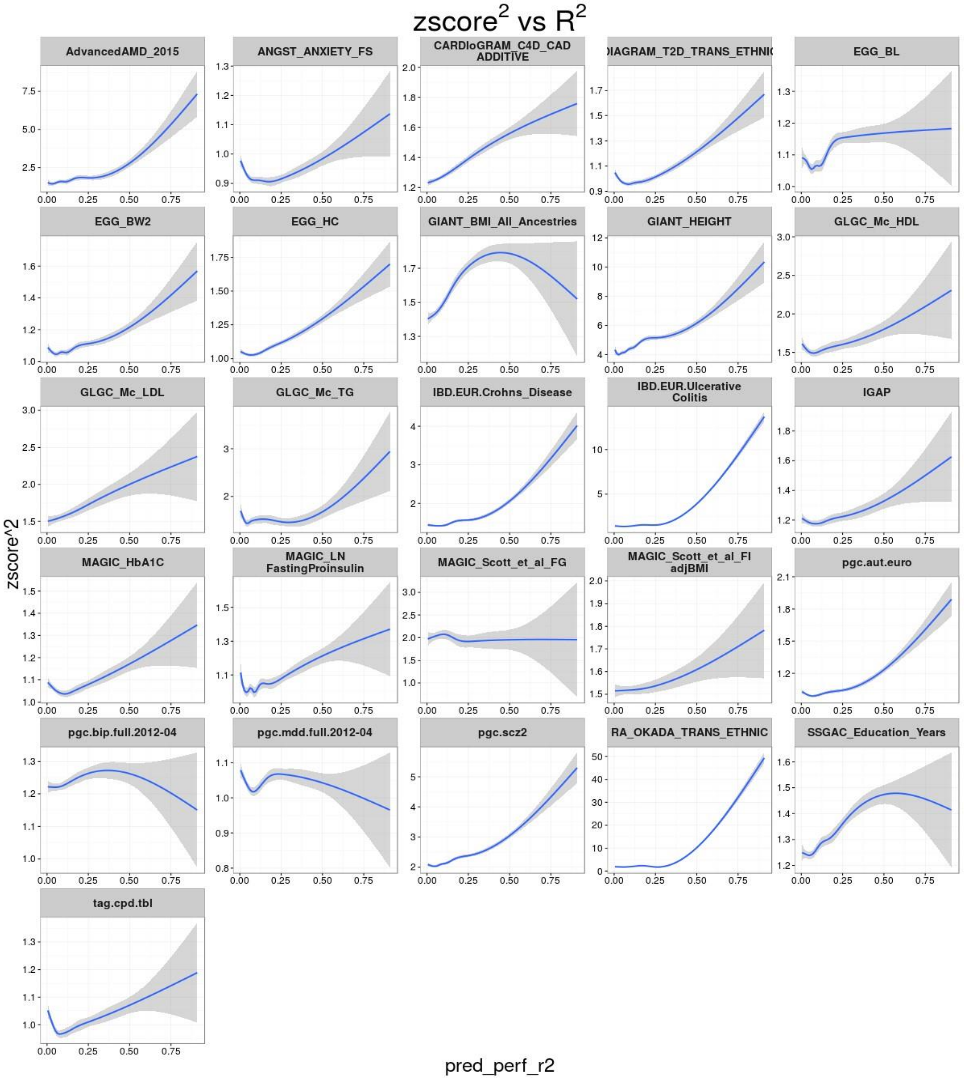

Supplementary Figure 1: Z-score^2 vs predicted performance R^2 by phenotype

When averaged across all genes and tissues within each phenotype the significance of the association tends to be more pronounced as R^2 (a measure of the genetic component) is larger. R^2 is the square of the correlation between predicted and observed expression levels in the training set, evaluated in a cross validated manner. The blue line depicts a Generalized Additive Model smoothing of the data. The gray shaded area depicts the 95% confidence interval of a linear model through each interpolated point.

Phenotype Abbreviation: AdvancedAMD_2015: Age-related Macular Degeneration; ANGST_ANXIETY_FS: Anxiety (Factor Score); CARDIoGRAM_C4D_CAD_ADDITIVE: Coronary Artery Disease (Additive Model); DIAGRAM_T2D_TRANS_ETHNIC: Type 2 Diabetes; EGG_BL: Birth Length; EGG_BW2: Birth Weight; EGG_HC: Hip Circumference; GIANT_BMI_All_Ancestries: Body Mass Index; GIANT_HEIGHT: Height; GLGC_Mc_HDL: High-Density Lipoprotein; GLGC_Mc_LDL: Low-Density Lipoprotein; GLGC_Mc_TG: Triglycerides; IBD.EUR.Crohns_Disease: Crohn's Disease; IBD.EUR.Ulcerative_Colitis: Ulcerative Colitis; IGAP: Alzheimer's Disease; MAGIC_HbA1C: Glycated Haemogoblin; MAGIC_LN_FastingProinsulin: Fasting Proinsulin; MAGIC_Scott_et_al_FG: Fasting Glucose; MAGIC_Scott_et_al_FI_adjBMI: Fasting Insulin (BMI Adjusted); pgc.aut.euro: Autistic Spectrum Disorder; pgc.bip.full.2012-04: Bipolar Disorder; pgc.mdd.full.2012-04: Major Depressive Disorder; pgc.scz2: Schizoprenia; RA_OKADA_TRANS_ETHNIC: Rheumatoid Arthritis; SSGAC_Education_Years: Education Years; tag.cpd.tbl: Cigarettes per day

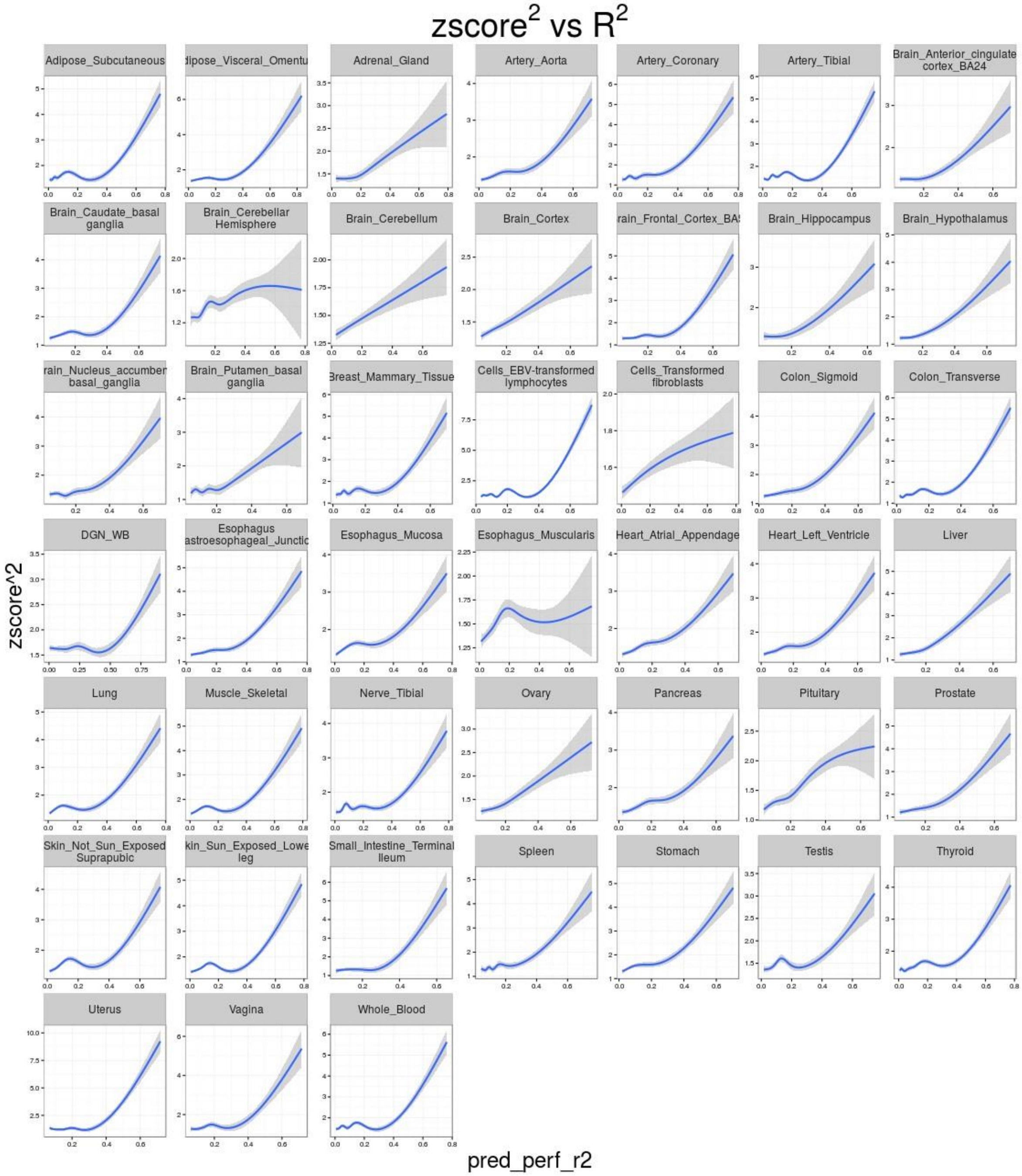

Supplementary Figure 2: Z-score^2 vs predicted performance R^2 by tissue

When averaged across all genes and phenotypes within each tissue the significance of the association tends to be more pronounced as R^2 (a measure of the genetic component) is larger. R^2 is the square of the correlation between predicted and observed expression levels in the training set, evaluated in a cross validated manner. The blue line depicts a Generalized Additive Model smoothing of the data. The gray shaded area depicts the 95% confidence interval of a linear model through each interpolated point.

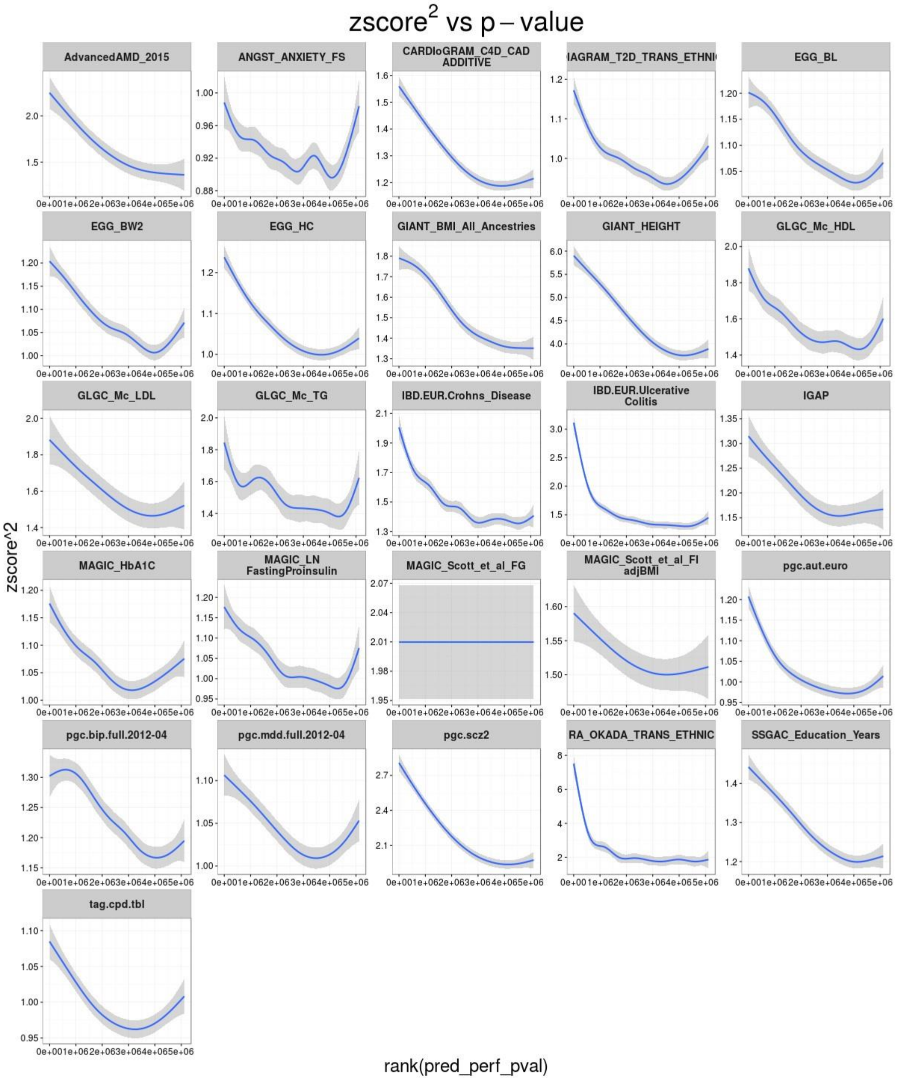

Supplementary Figure 3: Z-score^2 vs predicted performance p-value by phenotype

When averaged across all genes and tissues within each phenotype the significance of the association tends to be more pronounced as the cross validated prediction is more significantly associated with the observed expression. Prediction p-values (or prediction performance p-values) are computed (cross validated) as the p-values of the correlation between predicted and observed expression levels in the training set under the null hypothesis of no correlation. The blue line depicts a Generalized Additive Model smoothing of the data. The gray shaded area depicts the 95% confidence interval of a linear model through each interpolated point.

Phenotype Abbreviation: AdvancedAMD_2015: Age-related Macular Degeneration; ANGST_ANXIETY_FS: Anxiety (Factor Score); CARDIoGRAM_C4D_CAD_ADDITIVE: Coronary Artery Disease (Additive Model); DIAGRAM_T2D_TRANS_ETHNIC: Type 2 Diabetes; EGG_BL: Birth Length; EGG_BW2: Birth Weight; EGG_HC: Hip Circumference; GIANT_BMI_All_Ancestries: Body Mass Index; GIANT_HEIGHT: Height; GLGC_Mc_HDL: High-Density Lipoprotein; GLGC_Mc_LDL: Low-Density Lipoprotein; GLGC_Mc_TG: Triglycerides; IBD.EUR.Crohns_Disease: Crohn's Disease; IBD.EUR.Ulcerative_Colitis: Ulcerative Colitis; IGAP: Alzheimer's Disease; MAGIC_HbA1C: Glycated Haemogoblin; MAGIC_LN_FastingProinsulin: Fasting Proinsulin; MAGIC_Scott_et_al_FG: Fasting Glucose; MAGIC_Scott_et_al_FI_adjBMI: Fasting Insulin (BMI Adjusted); pgc.aut.euro: Autistic Spectrum Disorder; pgc.bip.full.2012-04: Bipolar Disorder; pgc.mdd.full.2012-04: Major Depressive Disorder; pgc.scz2: Schizoprenia; RA_OKADA_TRANS_ETHNIC: Rheumatoid Arthritis; SSGAC_Education_Years: Education Years; tag.cpd.tbl: Cigarettes per day

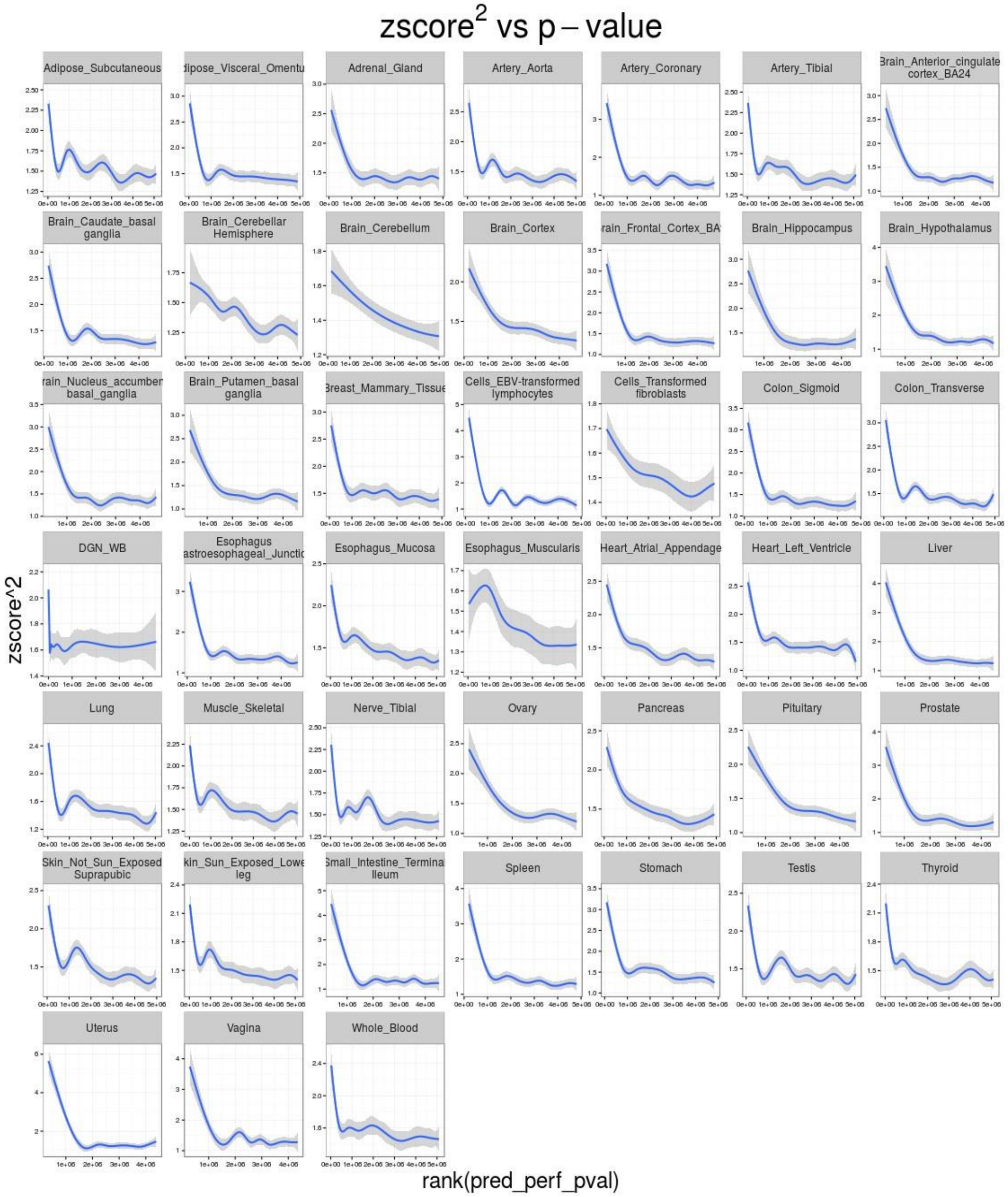

Supplementary Figure 4: Z-score^2 vs predicted performance p-value by tissue

When averaged across all genes and phenotypes within each tissue the significance of the association tends to be more pronounced as the cross validated prediction is more significantly associated with the observed expression. Prediction p-values (or prediction performance p-values) are computed (cross validated) as the p-values of the correlation between predicted and observed expression levels in the training set under the null hypothesis of no correlation. The blue line is a Generalized Additive Model smoothing of the data. The gray shaded area depicts the 95% confidence interval of a linear model through each interpolated point.

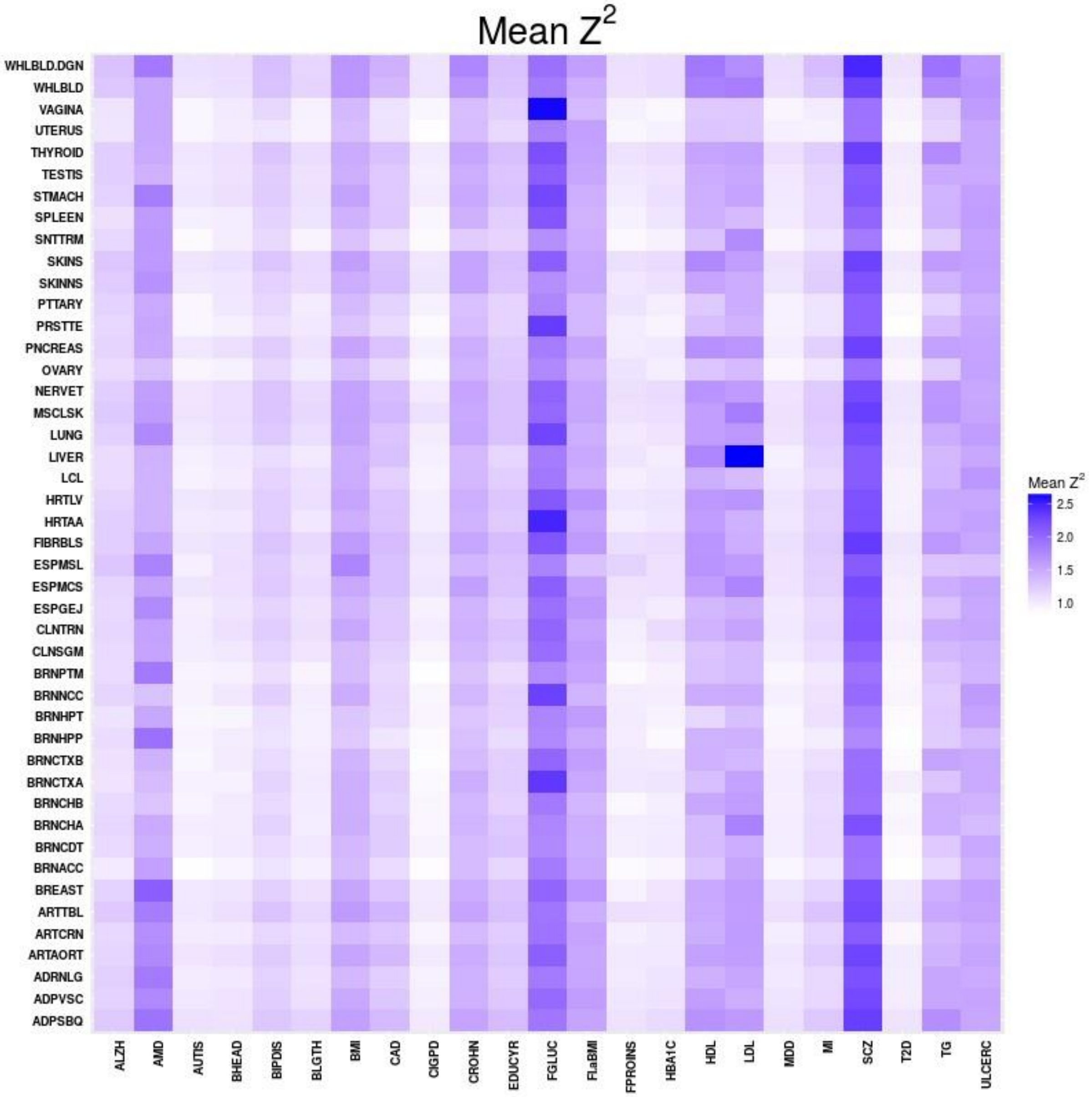

Supplementary Figure 5: Average enrichment of significant genes by tissues

This figure shows the average square of the Z-scores (effect size/standard error) of the association between the genetic component of gene expression levels and phenotype.

Phenotype abbreviations: CIGPD (cigarettes per day), BMI (body mass index), FGLUC (fasting glucose), T2D (type 2 diabetes), CAD (coronary artery disease), LDL (low-density lipoprotein cholesterol), TG (triglycerides), RA (rheumatoid arthritis), ALZH (alzheimer's disease), HDL (high-density lipoprotein cholesterol), CROHN (Crohn's disease), ULCERC (ulcerative colitis), HEIGHT, BHEAD (birth head circumference), BLGTH (birth length), BWEIG (birth weight), AUTIS (autism), EDUCYR (education years), SCZ (schizophrenia), AMD (age-related macular degeneration), ANX (anxiety), HBA1C (Hemogoblin A1C), FPROINS (fasting proinsulin), FI.aBMI (fasting insuline adjusted for BMI), MDD (major depressive disorder), BIPDIS (bipolar disorder).

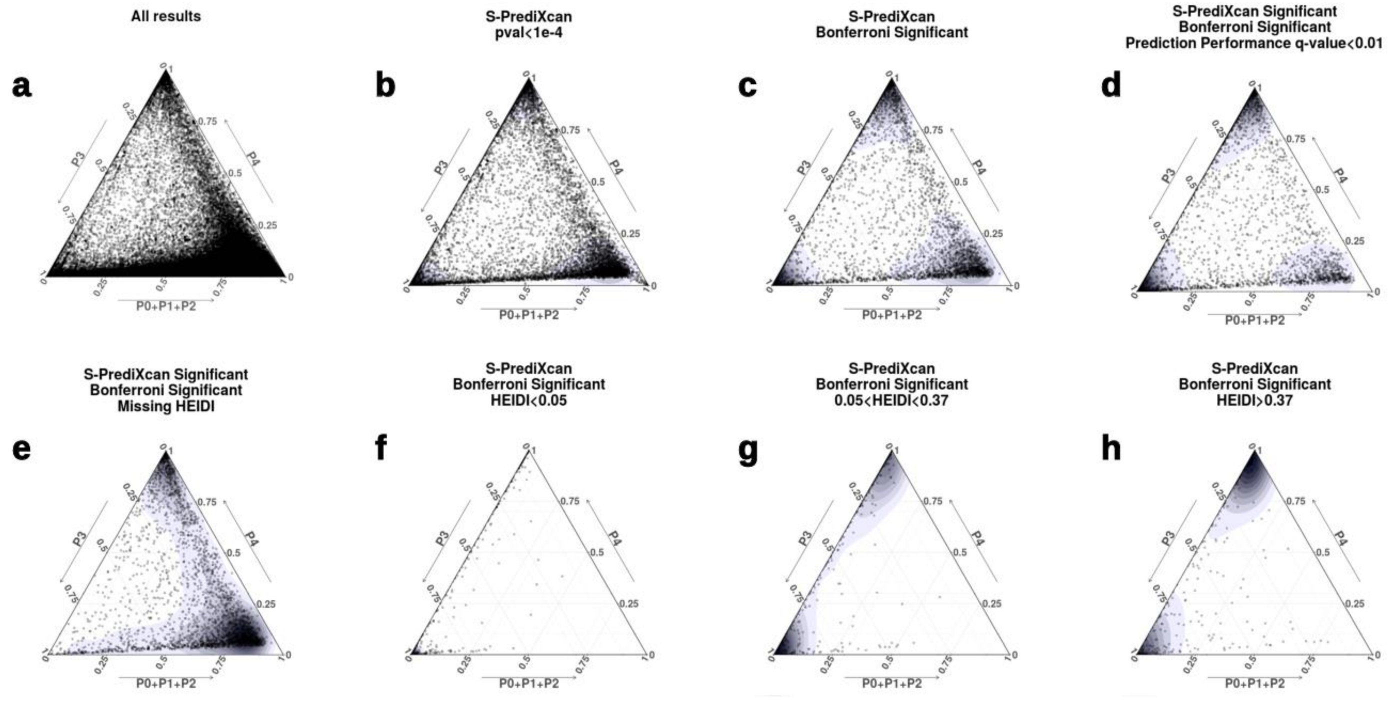

Supplementary Figure 6: Colocalization status of S-PrediXcan results for Height phenotype across all tissues

These ternary plots [10] constrain the values such that the sum of the probabilities is 1. All points in a horizontal line have the same probability of 'colocalized' GWAS and eQTL signals (P4), points on a line parallel to the right side of the triangle (NW to SE) have the same probability of'Independent signals' (P3), and lines parallel to the left side of the triangle (NE to SW) correspond to constant P1+P2+P3. Within each triangle: Top vertex corresponds to high probability of colocalization (P4>0.5), lower left vertex to probability of independent signals (P3>0.5), and lower right vertex corresponds to genes without enough power to determine or reject colocalization. **Panel a** shows that most the genes fall in the 'undetermined' region. When only significant S-PrediXcan associated genes are shown (**Panel b**:p< $1e-4$ & **Panel c**:p< $1e-6$), three peaks in each of the regions emerge (interpreted as 'colocalized', 'distinct', 'undetermined'). **Panel d** shows that when genes with low prediction performance are excluded, the 'undetermined' peak significantly diminishes. **Panel e** shows the COLOC probabilities for genes for which HEIDI returned no values. There is a significant peak in the undetermined region, but the density is still significant in other regions. **Panel f** shows genes that have significant HEIDI p-values, evidence of heterogeneity. As expected genes cluster mostly near probability of independent signals. **Panel h** shows genes that have non significant HEIDI p-value. Overall, HEIDI and COLOC tend to agree, although there is a sizable number of cases where the two methods will disagree. Unlike COLOC results, HEIDI does not partition the genes into distinct clusters and an arbitrary cutoff p-value has to be chosen.

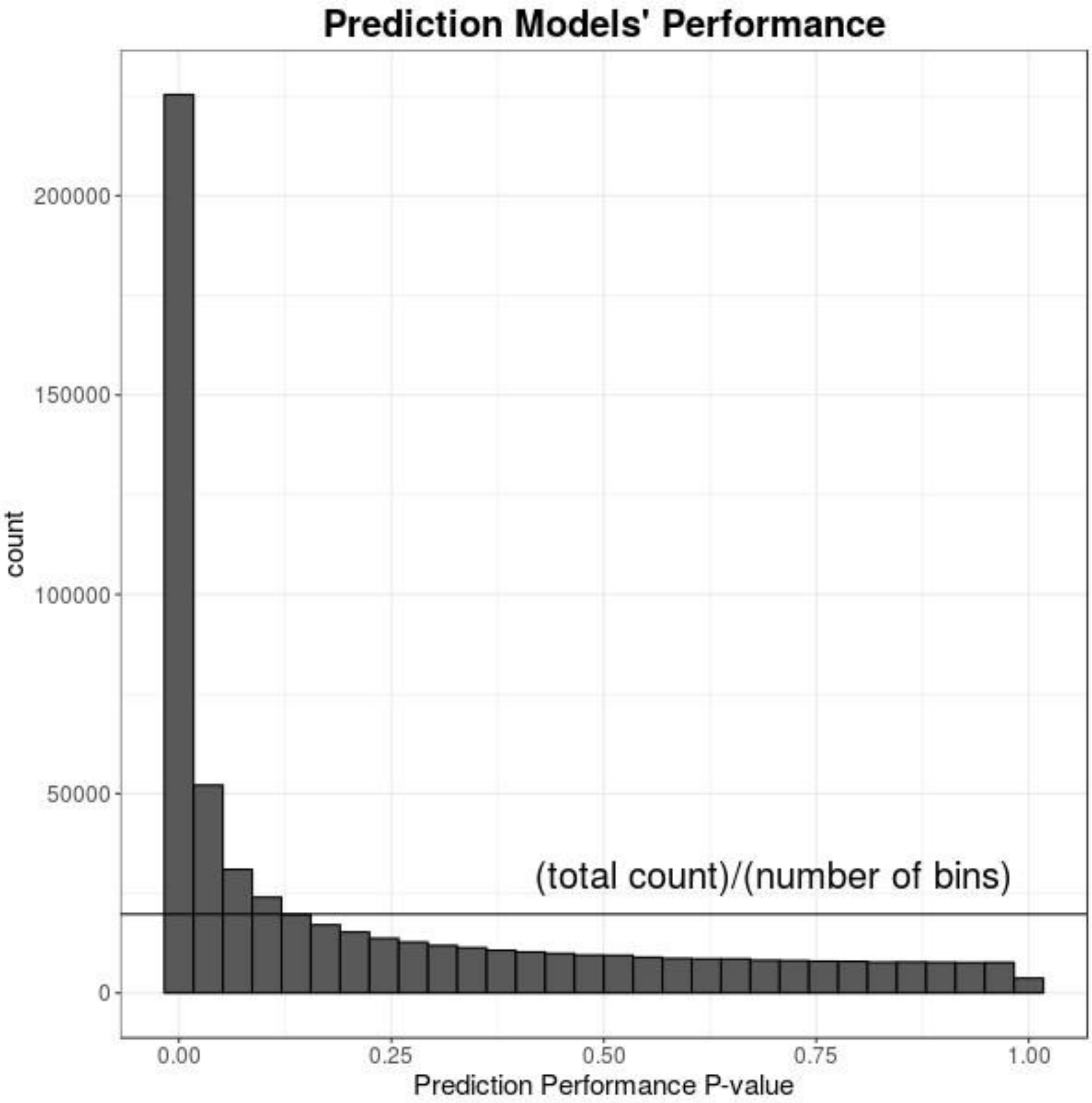

Supplementary Figure 7: Histogram of prediction performance p-value
This figure shows p-values of the correlation between predicted and observed expression levels in the training set. Prediction p-values (or prediction performance p-values) are computed (cross validated) as the p-values of the correlation between predicted and observed expression levels in the training set under the null hypothesis of no correlation.

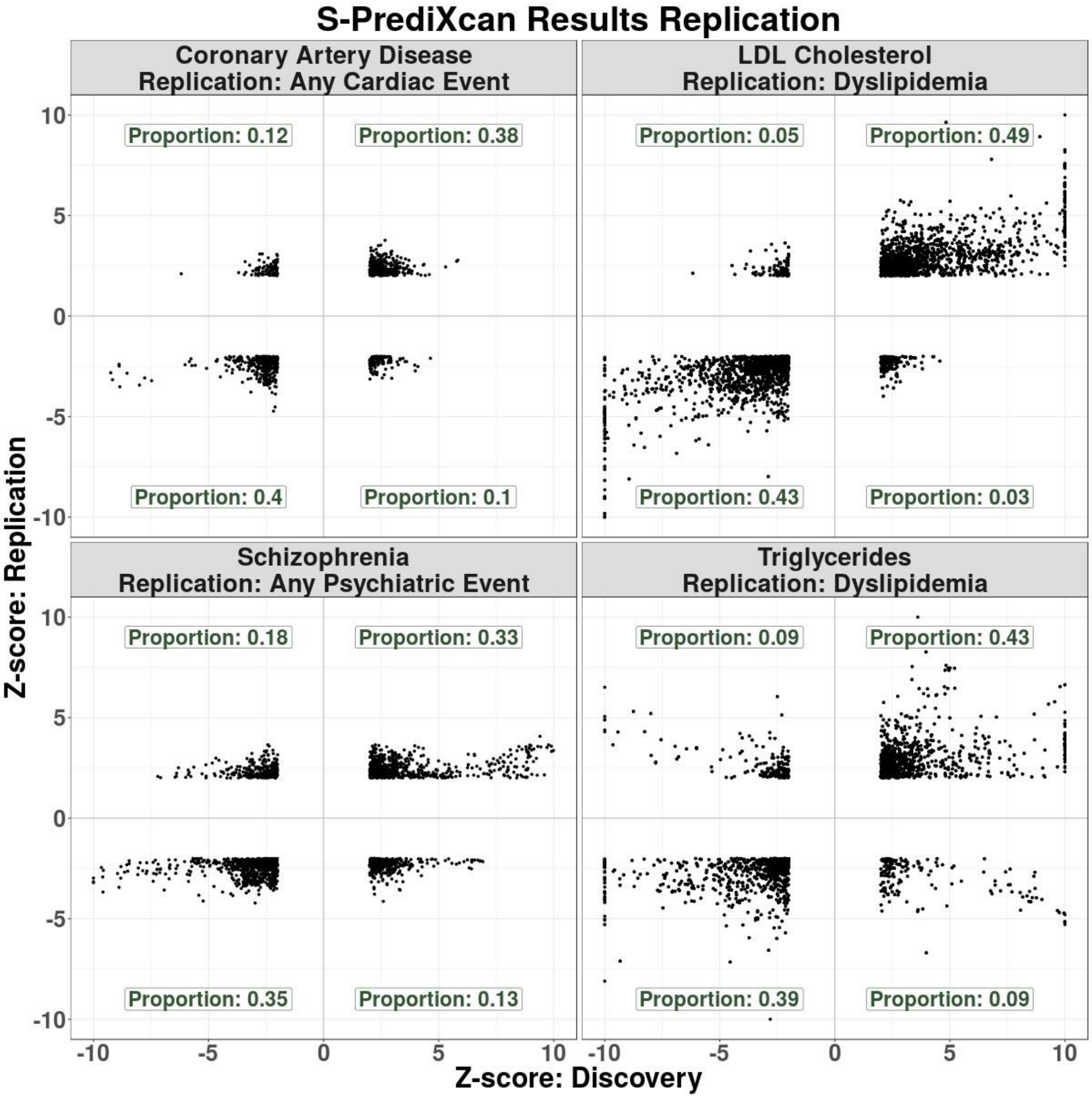

Supplementary Figure 8: Comparison of discovery and replication Z-scores

This figure shows the Z-scores of the discovery phenotype and the matched replication phenotype in GERA. The proportion of concordant direction of effects far exceeds the one with discordant direction of effects. Coronary artery disease has 77% of gene-tissue associations in the same direction of effects as ‘Any cardiac event’ in GERA. LDL cholesterol shows 92% concordance, TG shows 81%, and schizophrenia shows 67% concordance. Large Z-scores were thresholded to 10 to ease visualization. Proportions in each quadrant were computed excluding Z-scores with magnitude smaller than 2 to filter out noise.

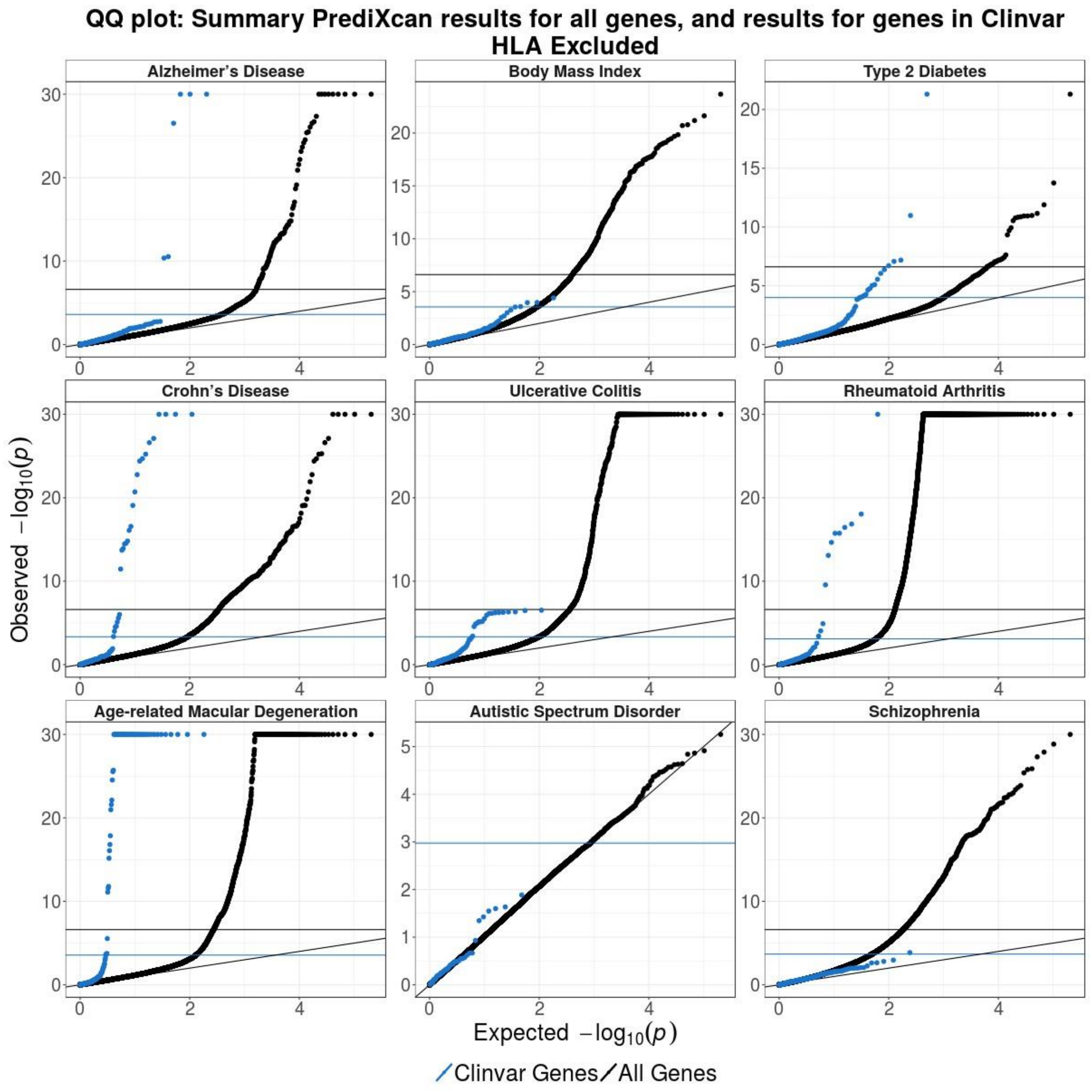

Supplementary Figure 9: ClinVar enrichment of S-PrediXcan associations, excluding genes in the HLA region
Blue circles correspond to the QQ plot of genes in ClinVar that were annotated with the phenotype and black circles correspond to all genes. Genes in the HLA region were excluded because of their complex LD structure, to verify the enrichment robustness. Rheumatoid Arthritis is the only phenotype that experienced a noticeable change, but still displayed significant enrichment.

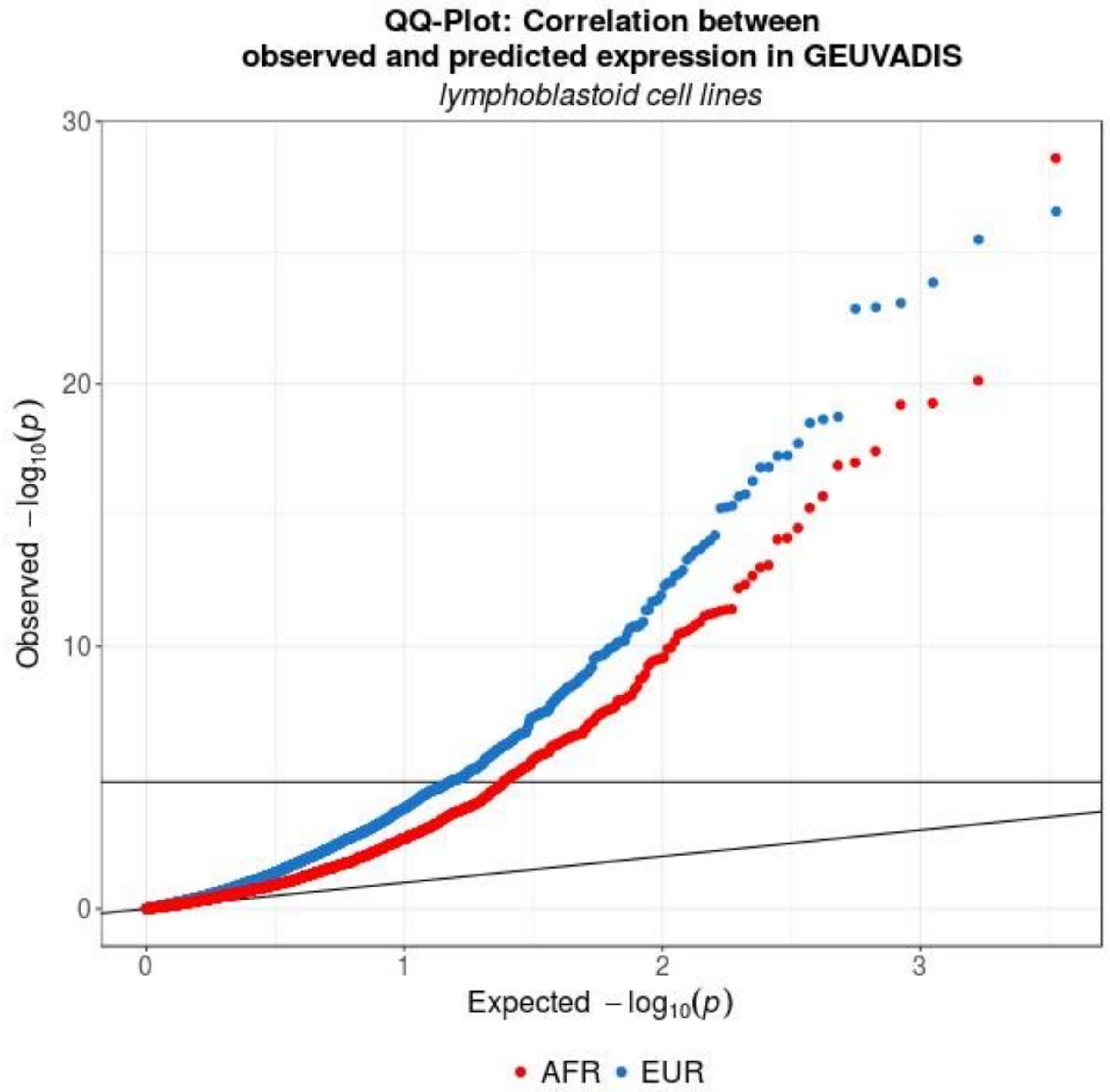

Supplementary Figure 10: Robustness of prediction across populations
Expression was predicted using prediction models trained on GTEx EBV transformed lymphocytes, with mostly European samples. Blue dots display the QQ plot of p-values of the correlation between predicted and observed gene expression levels in 77 European individuals from GEUVADIS [9]. Red dots correspond to the p-values of the correlation for 77 African individuals from GEUVADIS. There is only a small decrease in prediction performance in Africans compared to Europeans. Prediction with other tissue models showed entirely similar behavior.

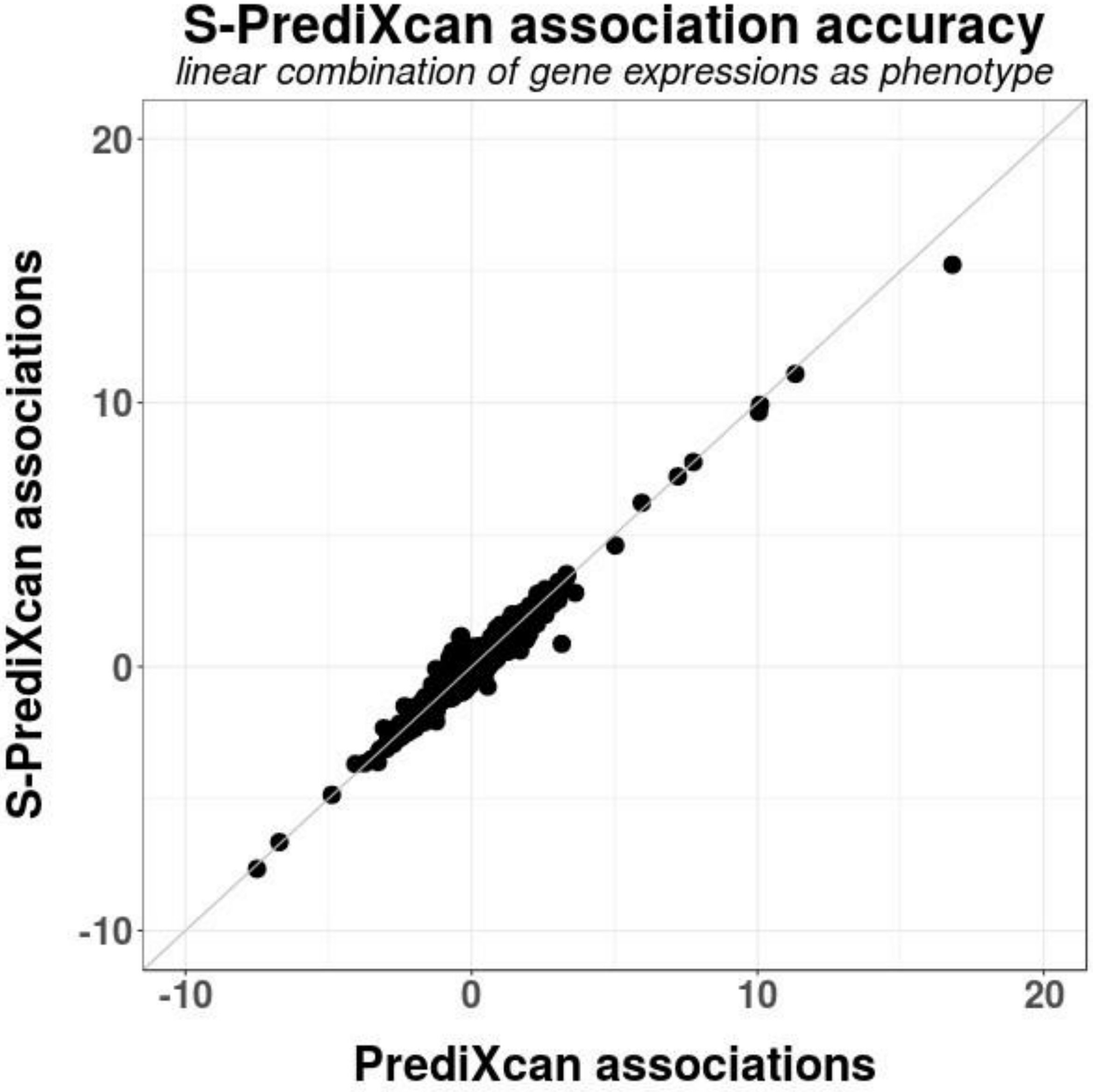

Supplementary Figure 11: S-PrediXcan Associations for a simulated phenotype under the alternative hypothesis

This figure illustrates S-PrediXcan's performance when the alternative hypothesis is true (i.e. the trait depends linearly on gene expression). We predicted gene expression on European individuals from the 1000 Genomes Project, using a model trained on GTEx Whole Blood study. We selected three genes (*SCYL3*, *MUSTN1*, *GCLC*) and built a phenotype according to $Y = 6T_{SCYL3} + T_{GLGC} + 4T_{MUSTN1} + \epsilon$, where T_X is predicted expression for gene X and ϵ is random noise sampled from a normal $N(0,1)$ distribution. The predicted expression component had standard deviation 1.46, so the noise is comparable to the signal.

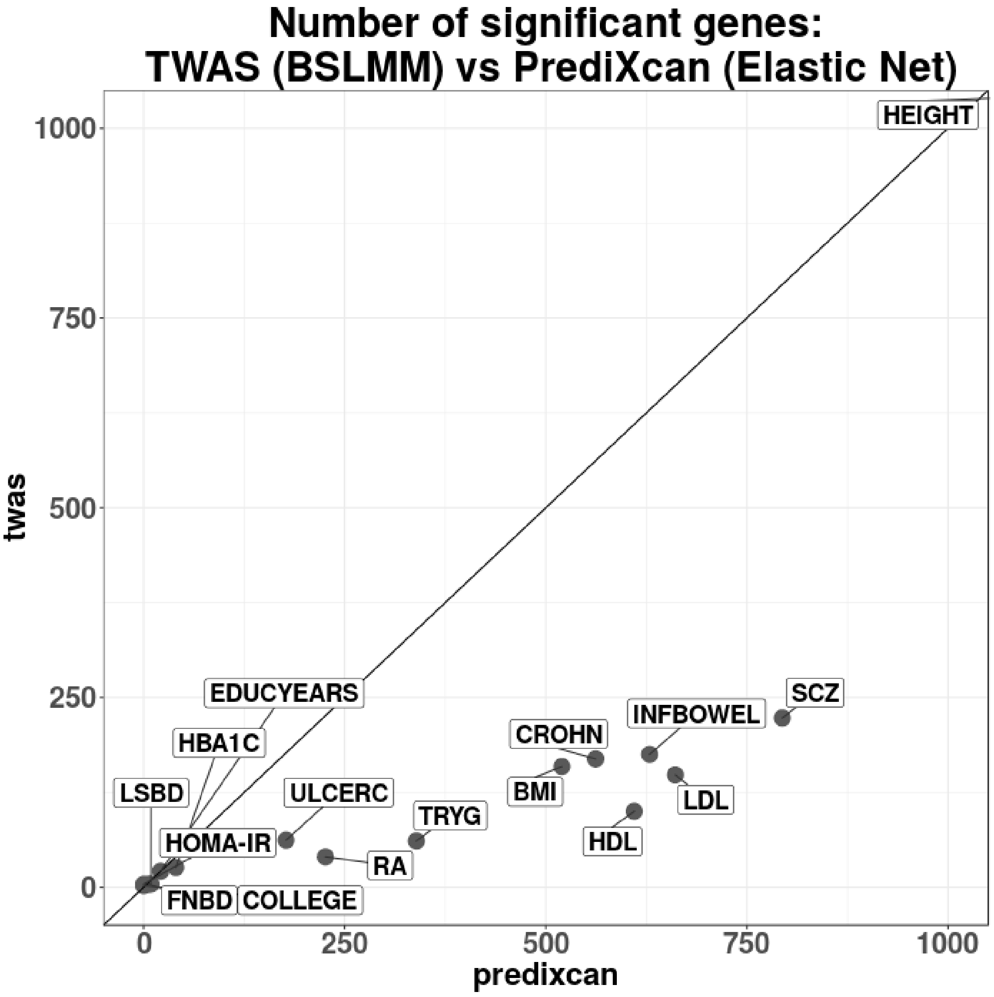

Supplementary Figure 12: Comparison of number of significant results between PrediXcan and TWAS

This figure contains results from TWAS and PrediXcan for the phenotypes and tissues reported in [24]. Notice that Mancuso et al filtered out genes with low GCTA heritability, which we have shown to underestimate the heritability measure h^2 [27]. This results in much smaller number of genes tested with TWAS than with PrediXcan. This in turn explains the smaller number of significant genes in TWAS despite the fact that when genes are tested, the significance of the two methods is similar as seen in Fig. 4b. Height trait is not shown for visualization purposes, but also exhibited this behavior.

Phenotype Abbreviation: Femoral Neck Bone Density (FNBD), Lumbar Spine Bone Density (LSBD), Body Mass Index (BMI), Height (HEIGHT), Low-Density Lipoprotein Cholesterol (LDL), High-Density Lipoprotein Cholesterol (HDL), Tryglicerides (TRYG), Crohn's Disease (CROHN), Inflammatory Bowel's Disease (INFBOWEL), Ulcerative Colitis (ULCERC), Hemogoblin Levels (HBA1C) HOMA Insulin Response (HOMA-IR) Schizophrenia (SCZ), Rheumatoid Arthritis (RA), College Completion (COLLEGE), Education Years (EDUCYEARS)

Supplementary Tables

Supplementary Table 1: Summary of Colocalization for S-PrediXcan Associations for selected phenotypes.
Column 'P4' lists the number of gene/tissue pairs that fall in the 'colocalized' region ($P(H4) > 0.5$, blue in Fig. 3a, 'P3' corresponds to 'non colocalized' or 'independent signal' region ($P(H3) > 0.5$, orange in Fig. 3a), 'undetermined' corresponds to region without strong evidence of either colocalization or non colocalization (gray in Fig. 3a), and the 'missing' column lists gene/tissue pairs for which colocalization yielded NA.

phenotype	total	P4	%	P3	%	undetermined	%	missing	%
Alzheimer's Disease	124	7	5.6%	44	35.5%	65	52.4%	8	6.5%
Bipolar Disorder	13	12	92.3%	0	0.0%	1	7.7%	0	0.0%
Birth Length	7	6	85.7%	0	0.0%	0	0.0%	1	14.3%
Body Mass Index	508	281	55.3%	122	24.0%	79	15.6%	26	5.1%
Cigarettes per Day	23	4	17.4%	7	30.4%	10	43.5%	2	8.7%
Coronary Artery Disease	136	93	68.4%	14	10.3%	20	14.7%	9	6.6%
Crohn's Disease	607	314	51.7%	166	27.3%	84	13.8%	43	7.1%
Education Years	20	19	95.0%	0	0.0%	0	0.0%	1	5.0%
Fasting Glucose	542	91	16.8%	64	11.8%	350	64.6%	37	6.8%
Fasting Insulin adjusted for BMI	102	25	24.5%	8	7.8%	63	61.8%	6	5.9%
Fasting Proinsulin	187	10	5.3%	74	39.6%	91	48.7%	12	6.4%
HDL Cholesterol	821	264	32.2%	236	28.7%	251	30.6%	70	8.5%
Height	5840	1672	28.6%	2063	35.3%	1724	29.5%	381	6.5%
Hemogoblin Levels	69	13	18.8%	36	52.2%	17	24.6%	3	4.3%
LDL Cholesterol	825	219	26.5%	221	26.8%	342	41.5%	43	5.2%
Major Depressive Disorder	1	1	100.0%	0	0.0%	0	0.0%	0	0.0%
Myocardial Infarction	80	69	86.2%	1	1.2%	3	3.8%	7	8.7%
Rheumatoid Arthritis	1580	159	10.1%	1219	77.2%	103	6.5%	99	6.3%
Schizophrenia	1122	283	25.2%	515	45.9%	254	22.6%	70	6.2%
Triglicerids	709	161	22.7%	242	34.1%	255	36.0%	51	7.2%
Type 2 Diabetes	33	19	57.6%	4	12.1%	6	18.2%	4	12.1%
Ulcerative Colitis	565	74	13.1%	371	65.7%	96	17.0%	24	4.2%

Supplementary Table 2: S-PrediXcan Association yields results more significant than Top SNPs.

Genes associated by S-PrediXcan to Coronary Artery Disease GWAS where S-PrediXcan outperforms individual SNPs in a 2 Mb window around the gene.

Gene Name	Tissue	P-value	Top SNP in Region	Top SNP P-value
FES	Cells Transformed fibroblasts	1.23E-08	rs2521501	5.0E-08
FHL3	Skin Sun Exposed Lower leg	1.99E-07	rs28470722	9.84E-07
IP6K2	Adipose Subcutaneous	2.14-07	rs7623687	5.22E-07
LIPA	Lung	1.84-12	rs1412444	5.15E-12
LIPA	Whole Blood	1.67-14	rs1412444	5.15E-12
NT5C2	Testis	3.79-09	rs11191416	4.65E-09
TCF21	Adrenal Gland	1.93E-11	rs12202017	1.98E-11
TCF21	Nerve Tibial	7.19E-12	rs12202017	1.98E-11
TUBG2	Adipose Visceral Omentum	2.34E-07	rs72823056	1.5E-06
IL6R	Colon Transverse	2.31E-10	rs6689306	2.6E-09
PCSK9	Nerve Tibial	1.04E-08	rs11206510	2.34E-08
SNF8	Thyroid	2.20E-07	rs35895680	3.76E-07
SWAP70	Spleen	1.00-08	rs10840293	1.28E-08
FURIN	Artery Aorta	1.27E-08	rs2521501	5.01E-08
UTP11L	Artery Tibial	1.58E-07	rs28470722	9.84E-07

Supplementary Table 3: S-PrediXcan association results for *SORT1*

Association with LDL cholesterol, coronary artery disease, and myocardial infarction are shown for available tissue models. Liver shows the most significant association with all three phenotypes. Also liver is the tissue with the most active regulation of *SORT1* expression, with 49% of the expression explained by our genetic prediction model. This is expected given the importance of this tissue in liver metabolism and its mediating effect on cardiovascular disease. P-value is the significance of the association between predicted expression levels and the phenotype. Effect size is the change in the phenotype when there is a change of 1 standard deviation in the predicted expression. Pred.Perf.R2 column is the cross validated R^2 in the training set between observed and predicted expression level. This can also be interpreted as a lower bound of the heritability of the expression trait. Pred.Perf.Pvalues is the p-values of the correlation between predicted and observed expression. *Note that tissue models will be available only when regulation was sufficiently active to yield a significant genetic component for the gene. Full set of results can be queried in gene2pheno.org. See more details in Supplementary Data 4.

Gene	Phenotype	Effect Size	Pvalue	Tissue	Pred.Perf.R2	Pred.Perf.Pvalue	P3	P4
SORT1	CAD	-0.09	1.3e-17	Liver	0.49	1.2e-15	0.00	1.00
		-0.14	3.6e-07	Pancreas	0.11	2.5e-05	0.07	0.88
		-0.25	9.3e-04	DGN WB	0.02	8.3e-05		
		-0.06	8.7e-03	Esophagus Mucosa	0.05	4.1e-04		
		0.03	5.6e-02	Small Intestine Terminal Ileum	0.17	1.7e-04		
		-0.05	1.5e-01	Spleen	0.09	3.9e-03		
		-0.02	2.4e-01	Testis	0.18	3.8e-08		
		0.08	5.4e-01	Brain Hippocampus	0.09	7.5e-03		
		-0.00	5.8e-01	Brain Anterior cingulate cortex BA24	0.17	2.9e-04		
		0.01	8.9e-01	Breast Mammary Tissue	0.03	2.4e-02		
SORT1	Myocardial Infarction	-0.08	5.2e-12	Liver	0.49	1.2e-15	0.00	1.00
		-0.12	4.4e-05	Pancreas	0.11	2.5e-05	0.07	0.88
		-0.21	1.2e-02	DGN WB	0.02	8.3e-05		
		-0.05	2.8e-02	Esophagus Mucosa	0.05	4.1e-04		
		0.03	1.2e-01	Small Intestine Terminal Ileum	0.17	1.7e-04		
		-0.02	2.4e-01	Testis	0.18	3.8e-08		
		-0.01	2.9e-01	Brain Anterior cingulate cortex BA24	0.17	2.9e-04		
		-0.04	3.3e-01	Spleen	0.09	3.9e-03		
		0.01	5.3e-01	Pituitary	0.06	1.8e-02		
		0.04	8.2e-01	Brain Hippocampus	0.09	7.5e-03		
SORT1	LDL-C	-0.14	7.4e-183	Liver	0.49	1.2e-15	0.00	1.00
		-0.24	6.5e-96	Pancreas	0.11	2.5e-05	0.05	0.90
		-0.11	2.9e-31	Esophagus Mucosa	0.05	4.1e-04	0.28	0.41
		-0.34	2.8e-27	DGN WB	0.02	8.3e-05		
		0.36	5.9e-11	Brain Hippocampus	0.09	7.5e-03	0.10	0.06
		-0.08	3.6e-06	Spleen	0.09	3.9e-03	0.11	0.06
		-0.03	5.5e-04	Testis	0.18	3.8e-08	1.00	0.00
		0.02	2.9e-02	Small Intestine Terminal Ileum	0.17	1.7e-04		
		-0.01	1.5e-01	Brain Anterior cingulate cortex BA24	0.17	2.9e-04		
		-0.01	2.0e-01	Pituitary	0.06	1.8e-02		

Supplementary Table 4: S-PrediXcan association between *C4A* and schizophrenia for available tissue models

C4A is actively regulated across all tissues, with prediction R^2 ranging from 8% to 39%. Predicted expression levels of *C4A* are also significantly associated with schizophrenia risk uniformly across all tissues. P-value is the significance of the association between predicted expression levels and the phenotype. Effect size is the change in the phenotype when there is a change of 1 standard deviation in the predicted expression. Pred.Perf.R2 column is the cross validated R^2 in the training set between observed and predicted expression level. This can also be interpreted as a lower bound of the heritability of the expression trait. Pred.Perf.Pvalues is the p-values of the correlation between predicted and observed expression. P-values of 0.02 and 0.03 for the Brain Hippocampus and Cortex results should not be interpreted as not associated. Brain tissues have limited sample size which could be one of the reasons why this association is less significant than in other tissues. For example there is no significant eQTL for this gene in Brain Hippocampus and Cortex. By using a multi SNP model we obtain significant models even when single eQTL analysis does not produce significant results. *Note that tissue models will be available only when regulation was sufficiently active to yield a significant genetic component for the gene. Full set of results can be queried in gene2pheno.org. See more details in Supplementary Data 4.

Gene	Phenotype	Effect Size	Pvalue	Tissue	Pred.Perf.R2	Pred.Perf.Pvalue	P3	P4
C4A	Schizophrenia	0.15	2.3e-20	Pancreas	0.27	1.7e-11	0.06	0.94
		0.16	7.7e-20	Artery Aorta	0.23	6.1e-13	0.44	0.56
		0.12	1.5e-19	Testis	0.35	4.6e-16	0.06	0.94
		0.13	2.6e-19	Thyroid	0.28	3.6e-21	0.46	0.54
		0.12	6.8e-19	Heart Atrial Appendage	0.39	8.6e-19	0.69	0.31
		0.15	8.5e-19	Adipose Subcutaneous	0.22	8.8e-18	0.10	0.90
		0.22	9.3e-19	Colon Sigmoid	0.16	2.9e-06	0.15	0.83
		0.15	1.0e-18	Heart Left Ventricle	0.26	5.6e-14	0.12	0.88
		0.13	1.2e-18	Liver	0.38	1.9e-11	0.33	0.67
		0.19	2.0e-18	Cells EBV-transformed lymphocytes	0.23	7.3e-08	0.07	0.92
		0.15	2.2e-18	Stomach	0.30	6.2e-15	0.16	0.84
		0.34	3.5e-18	Brain Hypothalamus	0.09	5.3e-03	0.27	0.39
		0.15	1.0e-17	Lung	0.20	8.3e-15	0.07	0.93
		0.16	2.7e-17	Colon Transverse	0.20	8.6e-10	0.07	0.93
		0.18	3.7e-17	Muscle Skeletal	0.18	1.3e-17	0.10	0.90
		0.11	4.7e-17	Nerve Tibial	0.33	1.2e-23	0.19	0.81
		0.18	9.1e-17	Adipose Visceral Omentum	0.22	1.8e-11	0.08	0.92
		0.13	3.6e-16	Brain Putamen basal ganglia	0.19	4.6e-05	0.17	0.62
		0.21	4.0e-16	Artery Coronary	0.10	5.2e-04	0.37	0.48
		0.15	1.2e-15	Brain Frontal Cortex BA9	0.18	3.1e-05	0.12	0.87
		0.16	1.7e-15	Esophagus Gastroesophageal Junction	0.22	2.0e-08	0.13	0.87
		0.12	4.1e-15	Prostate	0.21	6.2e-06	0.42	0.34
		0.12	6.2e-15	Esophagus Mucosa	0.26	1.5e-17	0.06	0.94
		0.13	1.5e-14	Breast Mammary Tissue	0.26	2.4e-13	0.26	0.74
		0.14	2.5e-14	Skin Sun Exposed Lower leg	0.24	2.3e-19	0.11	0.89
		0.14	1.2e-13	Brain Cerebellum	0.23	2.8e-07	0.10	0.88
		0.12	6.3e-13	Whole Blood	0.20	1.9e-18	0.05	0.95
		0.10	2.2e-12	Brain Cerebellar Hemisphere	0.17	7.3e-05	0.23	0.76
		0.08	4.3e-12	Skin Not Sun Exposed Suprapubic	0.35	7.1e-20	0.22	0.78
		0.10	1.0e-11	Cells Transformed fibroblasts	0.23	2.8e-17	0.12	0.88
		0.14	6.8e-11	Adrenal Gland	0.17	1.8e-06	0.19	0.78
		0.09	3.2e-10	Artery Tibial	0.17	5.2e-13	0.18	0.82
		0.08	5.7e-10	Brain Caudate basal ganglia	0.13	2.9e-04	0.30	0.34
		0.11	1.1e-09	Uterus	0.10	7.2e-03	0.34	0.07
		0.08	5.4e-09	Spleen	0.28	1.0e-07	0.07	0.93
		0.06	3.0e-08	Brain Anterior cingulate cortex BA24	0.25	9.3e-06	0.31	0.42
		0.12	1.1e-04	Small Intestine Terminal Ileum	0.08	1.1e-02	0.39	0.16
		0.05	2.6e-04	Pituitary	0.14	2.8e-04	0.30	0.32
		0.03	2.1e-02	Brain Hippocampus	0.12	1.2e-03	NA	NA
		0.03	3.4e-02	Brain Cortex	0.10	1.8e-03	NA	NA

Supplementary Table 5: S-PrediXcan association results for *PCSK9*

Association with LDL cholesterol, coronary artery disease, and myocardial infarction are shown for available tissue models. The significant association between LDL-C and *PCSK9* in visceral fat is consistent with other reports [35] but the most significant association is found in tibial nerve. Tibial nerve was the most actively regulated tissue with 18% of the expression level of the gene being explained by our genetic prediction model (cross validated). Pvalue is the significance of the association between predicted expression levels and the phenotype. Effect size is the change in the phenotype when there is a change of 1 standard deviation in the predicted expression. Pred.Perf.R2 column is the cross validated R^2 in the training set between observed and predicted expression level. This can also be interpreted as a lower bound of the heritability of the expression trait. Pred.Perf.Pvalues is the p-values of the correlation between predicted and observed expression. Even though some of these p-values are above 0.05, the corresponding FDR was less than 0.05, on account of small value of π_0 (estimated proportion of null associations). Supplementary Fig. 7 illustrates this point. *Note that tissue models will be available only when regulation was sufficiently active to yield a significant genetic component for the gene. Full set of results can be queried in gene2pheno.org. See more details in Supplementary Data 4.

Gene	Phenotype	Effect Size	Pvalue	Tissue	Pred.Perf.R2	Pred.Perf.Pvalue	P3	P4
PCSK9	CAD	0.13	1.0e-08	Nerve Tibial	0.18	1.5e-12	0.01	0.99
		0.49	4.1e-07	Lung	0.01	1.0e-01	0.01	0.98
		0.35	4.6e-05	Whole Blood	0.01	1.2e-01	0.09	0.84
		0.10	4.5e-03	Testis	0.04	9.1e-03		
		-0.17	1.2e-02	Colon Transverse	0.02	5.4e-02		
		0.07	2.0e-02	Adipose Visceral Omentum	0.06	1.1e-03		
		0.04	3.9e-02	Brain Cerebellum	0.07	6.1e-03		
		-0.06	2.3e-01	Skin Sun Exposed Lower leg	0.01	8.0e-02		
		0.07	3.5e-01	Artery Tibial	0.02	9.6e-03		
		-0.02	4.8e-01	Vagina	0.08	1.2e-02		
		-0.15	6.0e-01	Artery Coronary	0.04	3.8e-02		
PCSK9	Myocardial Infarction	0.12	8.6e-07	Nerve Tibial	0.18	1.5e-12	0.01	0.98
		0.48	7.9e-06	Lung	0.01	1.0e-01	0.01	0.97
		0.34	3.0e-04	Whole Blood	0.01	1.2e-01	0.07	0.75
		0.09	2.1e-02	Testis	0.04	9.1e-03		
		-0.10	8.5e-02	Skin Sun Exposed Lower leg	0.01	8.0e-02		
		0.05	8.8e-02	Adipose Visceral Omentum	0.06	1.1e-03		
		-0.11	1.5e-01	Colon Transverse	0.02	5.4e-02		
		0.03	2.5e-01	Brain Cerebellum	0.07	6.1e-03		
		-0.01	5.8e-01	Brain Cortex	0.04	3.9e-02		
		0.03	7.2e-01	Artery Tibial	0.02	9.6e-03		
		-0.01	8.5e-01	Vagina	0.08	1.2e-02		
PCSK9	LDL-C	0.13	1.4e-27	Nerve Tibial	0.18	1.5e-12	0.15	0.85
		-0.28	5.1e-21	Colon Transverse	0.02	5.4e-02	0.13	0.11
		0.43	2.2e-13	Lung	0.01	1.0e-01	0.02	0.91
		0.13	2.4e-13	Adipose Visceral Omentum	0.06	1.1e-03	0.69	0.05
		-0.16	4.3e-12	Skin Sun Exposed Lower leg	0.01	8.0e-02	0.15	0.10
		0.39	1.1e-10	Whole Blood	0.01	1.2e-01	0.07	0.60
		0.06	2.5e-10	Brain Cerebellum	0.07	6.1e-03	0.14	0.26
		0.03	3.6e-03	Brain Cortex	0.04	3.9e-02		
		-0.02	4.4e-01	Vagina	0.08	1.2e-02		
		0.05	4.7e-01	Testis	0.04	9.1e-03		
		-0.10	5.1e-01	Artery Coronary	0.04	3.8e-02		

Supplementary Table 6: List of Tissue Models
protein cod. lists the number of genes in the training set for the tissue, # samples is the samples available with expression and genotype data, # signif. models lists the number of models that achieved cross validated prediction significance FDR lower than 5%.

Tissue	# protein cod.	# samples	# signif models models (FDR <.05)
Adipose Subcutaneous	15935	298	7249
Adipose Visceral Omentum	15790	185	4568
Adrenal Gland	15370	126	4174
Artery Aorta	15401	197	6182
Artery Coronary	15437	118	3222
Artery Tibial	15388	285	7121
Brain Anterior cingulate cortex BA24	15385	72	2559
Brain Caudate basal ganglia	15658	100	3544
Brain Cerebellar Hemisphere	15202	89	4068
Brain Cerebellum	15456	103	4995
Brain Cortex	15652	96	3558
Brain Frontal Cortex BA9	15547	92	3258
Brain Hippocampus	15628	81	2566
Brain Hypothalamus	15818	81	2451
Brain Nucleus accumbens basal ganglia	15636	93	3057
Brain Putamen basal ganglia	15374	82	2749
Breast Mammary Tissue	16188	183	4648
Cells EBV-transformed lymphocytes	13905	114	3660
Cells Transformed fibroblasts	14556	272	7609
Colon Sigmoid	15599	124	3720
Colon Transverse	16010	169	4788
Esophagus Gastroesophageal Junction	15364	127	3601
Esophagus Mucosa	15741	241	6889
Esophagus Muscularis	15556	218	6533
Heart Atrial Appendage	15242	159	4565
Heart Left Ventricle	14834	190	4858
Liver	14767	97	2759
Lung	16336	278	6564
Muscle Skeletal	14959	361	6563
Nerve Tibial	15998	256	8113
Ovary	15238	85	2880
Pancreas	15335	149	4931
Pituitary	16131	87	3335
Prostate	15994	87	2614
Skin Not Sun Exposed Suprapubic	16110	196	5633
Skin Sun Exposed Lower leg	16259	302	7567
Small Intestine Terminal Ileum	15872	77	2613
Spleen	15371	89	3715
Stomach	15989	170	4096
Testis	17683	157	7043
Thyroid	16193	278	8026
Uterus	15164	70	2159
Vagina	15715	79	2041
Whole Blood	14858	338	6650

Supplementary References

1. Battle A, Mostafavi S, Zhu X, Potash JB, Weissman MM, McCormick C, et al. Characterizing the genetic basis of transcriptome diversity through RNA-sequencing of 922 individuals. Genome Research. 2014;24(1):14–24.

2. Wheeler HE, Shah KP, Brenner J, Garcia T, Aquino-Michaels K, Cox NJ, et al. Survey of the Heritability and Sparse Architecture of Gene Expression Traits across Human Tissues. PLoS Genetics. 2016;12(11).

3. Im HK, Gamazon ER, Stark AL, Huang RS, Cox NJ, Dolan ME. Mixed effects modeling of proliferation rates in cell-based models: Consequence for pharmacogenomics and Cancer. PLoS Genetics. 2012;8(2).

4. Zhou X, Carbonetto P, Stephens M. Polygenic Modeling with Bayesian Sparse Linear Mixed Models. PLoS Genetics. 2013;9(2).

5. Nica AC, Montgomery SB, Dimas AS, Stranger BE, Beazley C, Barroso I, et al. Candidate causal regulatory effects by integration of expression QTLs with complex trait genetic associations. PLoS Genetics. 2010;6(4).

6. Nicolae DL, Gamazon E, Zhang W, Duan S, Eileen Dolan M, Cox NJ. Trait-associated SNPs are more likely to be eQTLs: Annotation to enhance discovery from GWAS. PLoS Genetics. 2010;6(4).

7. Li YI, van de Geijn B, Raj A, Knowles DA, Petti AA, Golan D, et al. RNA splicing is a primary link between genetic variation and disease. Science. 2016;352(6285):600–604. Available from: http://www.ncbi.nlm.nih.gov/pubmed/27126046.

8. Gamazon ER, Wheeler HE, Shah KP, Mozaffari SV, Aquino-Michaels K, Carroll RJ, et al. A genebased association method for mapping traits using reference transcriptome data. Nature genetics. 2015;47(9):1091–1098. Available from: http://dx.doi.org/10.1038/ng.3367.

9. Lappalainen T, Sammeth M, Friedländer MR, ’t Hoen PaC, Monlong J, Rivas Ma, et al. Transcriptome and genome sequencing uncovers functional variation in humans. Nature. 2013;501(7468):506–11. Available from: https://www.ncbi.nlm.nih.gov/pmc/articles/PMC3918453/.

10. Hamilton N. ggtern: An Extension to ’ggplot2’, for the Creation of Ternary Diagrams; 2016. R package version 2.2.0. Available from: https://CRAN.R-project.org/package=ggtern.
