# Supplementary material for: Exploring the phenotypic consequences of tissue specific gene expression variation inferred from GWAS summary statistics
Source: Nat Commun. 2018 May 8;9:1825. doi: 10.1038/s41467-018-03621-1 (PMC5940825; doi:10.1038/s41467-018-03621-1)
Supplement: Supplementary file 3 — Description of Additional Supplementary Files [file 41467_2018_3621_MOESM3_ESM.pdf]

Description of Additional Supplementary Files

File Name: Supplementary Data 1
Description: ClinVar genes with significant association in MetaXcan
Columns are: ClinVar disease name; gene2pheno.org phenotype name; gene name; p-value of the association; tissue where the model was trained.

List of Genome-wide Association Meta Analysis (GWAMA) Consortia and phenotypes
Columns are consortium name; study name; gene2pheno.org display name; link to pubmed entry if available; study sample size; study population; # of significant gene-tissue pairs; # of significant unique genes; # remaining after filtering prediction performance; # remaining after discarding high non-shared signals probability; # remaining after discarding undetermined results from last column.

File Name: Supplementary Data 3
Description: List of Significant Gene Association Results
Columns are consortium name; gene2pheno.org display name; study name; gene name; tissue; zscore; p-value; probability of 'undetermined' colocalization; probability of non-shared signals; probability of colocalized signals.

File Name: Supplementary Data 4
Description: MetaXcan results for *C4A, PCSK9, SORT1*
Columns are gene name; study name; gene2pheno.org display name; tissue; effect size; zscore; S-PrediXcan p-value; probability of no causal SNP in either GWAS or eQTL; probability of causal SNP only in GWAS; probability of causal SNP only in eQTL; probability of non-shared signals, probability of colocalized signals, prediction performance cross-validated R^2 , prediction performance cross-validated p-value; prediction performance false discovery ratio; number of SNPs used in S-PrediXcan, number of snps available in the model; SMR p-value; HEIDI probability; S-TWAS p-
